# Prevention of severe lung immunopathology associated with influenza infection through adeno-associated virus vector administration

**DOI:** 10.1186/s42826-023-00177-0

**Published:** 2023-10-30

**Authors:** Eun Ah Choi, Hi Jung Park, Sung Min Choi, Jae Il Lee, Kyeong Cheon Jung

**Affiliations:** 1 Graduate Course of Translational Medicine, Seoul National University College of Medicine, Seoul, 03080 Republic of Korea; 2 Transplantation Research Institute, Seoul National University College of Medicine, Seoul, 03080 Republic of Korea; 3 Department of Pathology, Seoul National University College of Medicine, Seoul, 03080 Republic of Korea; 4 Integrated Major in Innovative Medical Science, Seoul National University Graduate School, Seoul, 03080 Republic of Korea; 5 Department of Medicine, Seoul National University College of Medicine, Seoul, 03080 Republic of Korea

**Keywords:** Influenza A virus, Adeno-associated virus, Lung injury, Immunopathology, Inflammatory cells

## Abstract

**Background:**

Influenza A viruses (IAVs) have long posed a threat to humans, occasionally causing significant morbidity and mortality. The initial immune response is triggered by infected epithelial cells, alveolar macrophages and dendritic cells. However, an exaggerated innate immune response can result in severe lung injury and even host mortality. One notable pathology observed in hosts succumbing to severe influenza is the excessive influx of neutrophils and monocytes into the lung. In this study, we investigated a strategy for controlling lung immunopathology following severe influenza infection.

**Results:**

To evaluate the impact of innate immunity on influenza-associated lung injury, we employed CB17.SCID and NOD.SCID mice. NOD.SCID mice exhibited slower weight loss and longer survival than CB17.SCID mice following influenza infection. Lung inflammation was reduced in NOD.SCID mice compared to CB17.SCID mice. Bulk RNA sequencing analysis of lung tissue showed significant downregulation of 827 genes, and differentially expressed gene analysis indicated that the cytokine-cytokine receptor interaction pathway was predominantly downregulated in NOD.SCID mice. Interestingly, the expression of the *Cxcl14* gene was higher in the lungs of influenza-infected NOD.SCID mice than in CB17.SCID mice. Therefore, we induced overexpression of the *Cxcl14* gene in the lung using the adeno-associated virus 9 (AAV9)-vector system for target gene delivery. However, when we administered the AAV9 vector carrying the *Cxcl14* gene or a control AAV9 vector to BALB/c mice from both groups, the morbidity and mortality rates remained similar. Both groups exhibited lower morbidity and mortality than the naive group that did not receive the AAV9 vector prior to IAV infection, suggesting that the pre-administration of the AAV9 vector conferred protection against lethal influenza infection, irrespective of *Cxcl14* overexpression. Furthermore, we found that pre-inoculation of BALB/c mice with AAV9 attenuated the infiltration of trans-macrophages, neutrophils and monocytes in the lungs following IAV infection. Although there was no difference in lung viral titers between the naive group and the AAV9 pre-inoculated group, pre-inoculation with AAV9 conferred lung injury protection against lethal influenza infection in mice.

**Conclusions:**

Our study demonstrated that pre-inoculation with AAV9 prior to IAV infection protected mouse lungs from immunopathology by reducing the recruitment of inflammatory cells.

**Supplementary Information:**

The online version contains supplementary material available at 10.1186/s42826-023-00177-0.

## Background

Influenza A viruses (IAVs) are enveloped single-stranded RNA viruses with eight segmented genes and are characterized by hemagglutinin (HA) and neuraminidase (NA) surface glycoproteins, which are the primary targets of neutralizing antibodies [1, 2]. These viruses exhibit rapid evolution with point mutations in viral proteins, known as ‘antigenic drift’, that allow them to evade immunity conferred by previous infection or vaccination and cause annual outbreaks [2, 3]. Severe morbidity and mortality associated with pandemic IAVs primarily result from lung injury caused by severe immunopathology and secondary bacterial infection [4]. The major observed pathology in the lungs of hosts with severe influenza is the massive influx of neutrophils and monocytes [5]. The initial immune response to IAV infection is the secretion of interferons by infected epithelial cells, alveolar macrophages and dendritic cells, followed by the recruitment of inflammatory cells such as neutrophils, monocytes/macrophages and natural killer cells, and finally the activation of adaptive immune cells, which contribute to virus clearance and host recovery [6].

Neutrophils can play both beneficial and detrimental roles during influenza infection [7]. Studies have shown that depleting neutrophils prior to sublethal IAV infection in mice, results in uncontrolled viral growth and increased mortality, indicating a protective role for neutrophils. These findings were further supported by subsequent studies [7–9], which highlighted the production of the antimicrobial peptide mCRAMP by neutrophils and their ability to induce the release of IL-1β in alveolar macrophages [8]. Neutrophils also play a crucial role in directing the migration of influenza-specific CD8 T cells to the site of infection and maintaining CD8 T cell effector responses [9, 10]. However, another study demonstrated that a transcriptional signature of neutrophil accumulation in the lung during influenza infection could predict a lethal outcome [11]. In other words, attenuating rather than completely depleting neutrophils reduced mortality in mice, suggesting the need to dampen exaggerated neutrophilic inflammation to protect hosts from lethal influenza infection [11]. Upon exposure to IAVs, alveolar macrophages promote the recruitment of CCR2^+^Ly6C^hi^ inflammatory monocytes [6, 12]. These monocytes further differentiate into inflammatory monocyte-derived macrophages and inflammatory dendritic cells, which promote the recruitment of protective NK cells [6, 12]. However, prolonged recruitment of inflammatory monocytes has been reported to contribute to the lethality of severe influenza infection, and depleting these cells has been shown to attenuate lung injury [13, 14].

Adeno-associated virus (AAV) is a nonenveloped single-stranded DNA parvovirus with a genome size of approximately 4.8 kb. Due to its nonpathogenic nature, low immunogenicity and broad tropism, AAV has emerged as a valuable tool for delivering therapeutic genes with long-term expression in both animals and humans [15, 16]. Following successful trials in hemophilia patients over a decade ago [17], AAV vectors have been extensively studied in clinical trials targeting various genetic diseases. The AAV genome consists of replication (rep) and capsid (cap) genes, with the genomic DNA flanked by two inverted terminal repeats (ITRs) [17, 18]. AAV vectors are constructed by replacing most of the viral genome with expression cassettes consisting of a promoter, a gene of interest and a poly A tail [19]. Once integrated into host cells, 3′-ITR serves as a primer for host DNA polymerase, generating a self-complementary intermediate comprising both plus and minus DNA strands [17, 18]. Interestingly, a previous study suggested that both plus and minus strand RNAs could be copied from the self-complementary intermediate DNA within the cytoplasm of AAV-transduced cells, due to the inherent promoter activity of 5′- and 3′-ITRs [20]. This process results in the formation of double-stranded RNA (dsRNA), triggering the cytoplasmic dsRNA sensor MDA5 in host cells. Sensing dsRNA leads to the production of IFN-β, which subsequently suppresses transgene expression [20]. However, it remains undetermined whether in vivo gene transduction using AAV vectors can also influence other immune responses, such as the response to IAV infection.

In this study, we employed CB17.SCID and NOD.SCID mice to investigate a strategy for controlling lung immunopathology following severe influenza infection. Our findings revealed that NOD.SCID mice exhibited reduced morbidity and mortality compared to CB17.SCID mice, which was associated with attenuated infiltration of neutrophils and inflammatory monocytes. Notably, *Cxcl14* gene expression was higher in the infected lungs of NOD.SCID mice than that of CB17.SCID mice, in contrast to other inflammatory cytokines and chemokines. Based on these findings, we attempted to overexpress the *Cxcl14* gene in the lungs of mice using the AAV9 vector. Interestingly, mice receiving the AAV9 vector itself, independent of *Cxcl14* gene overexpression, exhibited significantly reduced mortality after IAV challenge compared to mice not receiving the AAV9 vector. Further analysis revealed that the increased survival was associated with decreased infiltration of neutrophils and trans-macrophages in the infected lungs. These results demonstrated the immunoregulatory potential of AAV9 vectors.

## Results

### Attenuated lung inflammation in NOD.SCID mice compared to CB17.SCID mice

**Fig. 1:**
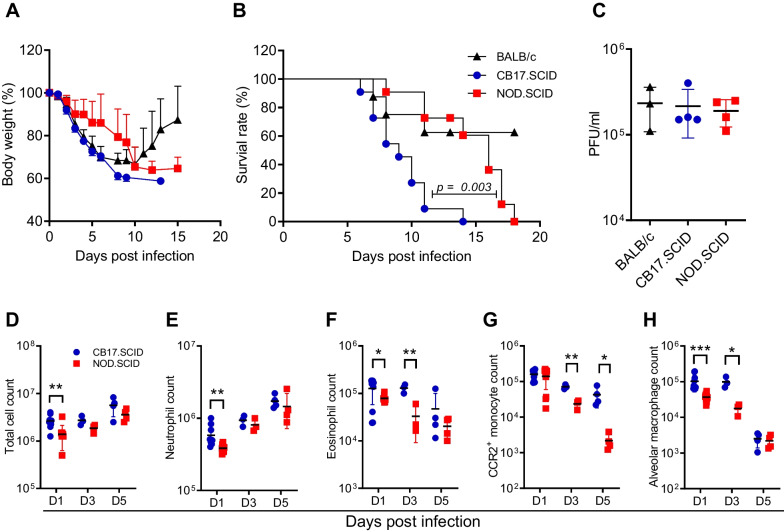
Improved prognosis in NOD.SCID mice than CB17.SCID mice after IAV infection. (**A**) Body weight and (**B**) mortality were monitored in BALB/c, CB17.SCID, and NOD.SCID mice after infection with IAV. Cumulative data from three independent experiments (n = 15 per group) show attenuated weight loss and prolonged survival in NOD.SCID mice compared to the CB17.SCID control. Survival rate differences were statistically significant as determined by the log-rank (Mantel-Cox) test. (**C**) On day 5 post-infection, lungs were harvested from IAV-infected mice, and the virus titer was measured by plaque assay. Cumulative data from three mouse groups (n = 5 per group) show no difference in virus titers between the groups. (**D**–**G**) Immune cells were extracted from the lungs of CB17.SCID and NOD.SCID mice on days 1, 3, and 5 post-infection, with the absolute number of each immune cell type determined through flow cytometric analysis. Comparative evaluation of total cell counts (**D**), neutrophils (**E**), eosinophils (**F**), CCR2^+^ inflammatory monocytes (**G**), and alveolar macrophages (**H**) between the two groups was carried out. Cumulative data from two groups of mice (n = 5 per group) show statistically significant differences in immune cell counts, analyzed using an unpaired t-test. Statistical significance is indicated as follows: *p < 0.05; **p < 0.04; ***p < 0.001

To evaluate the impact of innate and adaptive immunity on influenza-associated lung injury, we utilized BALB/c, CB17.SCID and NOD.SCID mice in our study. All three groups of mice were intranasally infected with a sublethal dose of IAV, and changes in body weight and mortality were monitored. Following influenza challenge, body weight progressively decreased in all mouse groups, with no difference in weight loss observed between BABL/c and CB17.SCID mice during the first six days (Fig. 1A). Then, BALB/c mice regained body weight and ultimately survived, while CB17.SCID mice failed to recover, resulting in 100% mortality as expected (Fig. 1B). In contrast, NOD.SCID mice exhibited slower weight loss and significantly prolonged survival compared to CB17.SCID mice (Fig. 1A, B).

These findings raised two possibilities: reduced viral replication or attenuated lung inflammation in NOD.SCID mice compared to the other groups. To assess the first possibility, virus titers were measured using a plaque assay on homogenized lung tissue on day 5 post-infection. As shown in Fig. 1C, no difference was observed in viral titers among the three mouse groups. Subsequently, we evaluated the inflammatory response in infected lungs on day 1, day 3, and day 5 post-infection through flow cytometric analysis, which revealed attenuated infiltration of inflammatory cells in NOD.SCID lungs compared to CB17.SCID lungs (Fig. 1D). Notably, distinct kinetics were observed for granulocytes and inflammatory monocytes. The number of neutrophils and eosinophils was significantly lower in NOD.SCID mice than in CB17.SCID controls on day 1 and day 3 post-infection, and the difference between the two groups gradually diminished during the course of lung inflammation (Fig. 1E, F). Conversely, CCR2^+^ inflammatory monocytes, which are closely associated with the severity of inflammation in IAV infection [13], initially infiltrated the lungs of both groups in comparable quantities. However, the number of infiltrating cells declined more rapidly in NOD.SCID lung compared to the CB17.SCID control (Fig. 1G). These results are consistent with previous reports that inflammatory monocytes and neutrophils contribute to lethal lung injury in influenza and SARS-CoV infections [13, 21]. Additionally, studies have shown that CCR2 blockade reduces lung pathology associated with influenza infection and partially suppresses the neutrophil response in mice exposed to lethal IAV strains [11, 22]. Taken together, the attenuated infiltration of inflammatory monocytes and neutrophils in NOD.SCID mice may contribute to their prolonged survival.

In contrast to granulocytes and inflammatory monocytes, alveolar macrophages, which are long-lived in the lung alveolar space after birth, are typically depleted following influenza infection and replaced by bone marrow-derived trans-macrophages [14, 23]. Consistent with this, the number of alveolar macrophages progressively decreased in the infected lungs of both groups, although NOD.SCID mice exhibited a lower number of cells in the early stages of infection compared to CB17.SCID mice (Fig. 1H). Alveolar macrophages are known to play a protective role in influenza infection and have been reported to protect alveolar epithelial cells against IAV infection [24, 25]. Thus, alveolar macrophages may not be the primary contributors to early lethality in CB17.SCID mice.

### Higher *Cxcl14* gene expression in the lungs of influenza-infected NOD.SCID mice compared to CB17.SCID mice

**Fig. 2:**
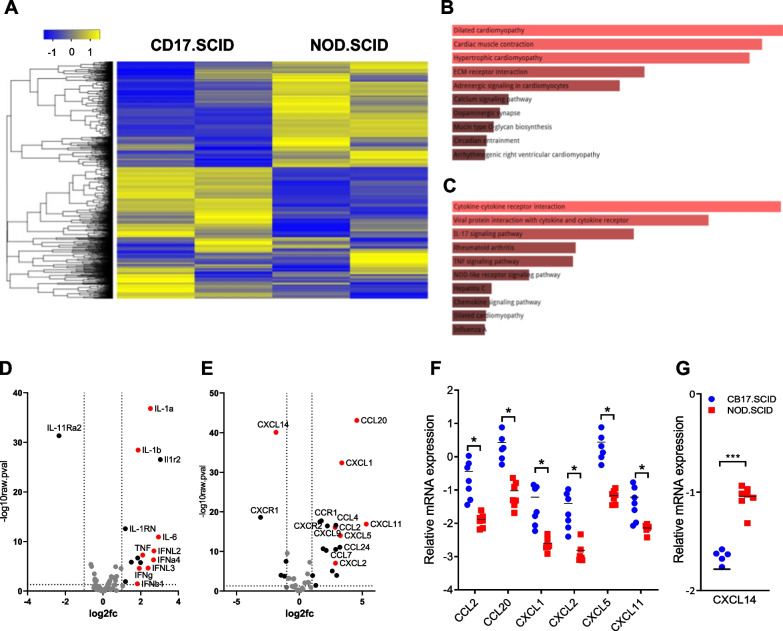
Comparative analysis of gene expression profiles and selected chemokine genes in IAV-infected lungs of CB17.SCID and NOD.SCID mice. Total mRNA was extracted from the lungs on day 1 post-infection, and bulk RNA sequencing analysis was conducted. (**A**) A heatmap depicting differentially expressed genes between CB17.SCID and NOD.SCID mice (n = 2 per group). Gene sets enriched in NOD.SCID (**B**) and CB17.SCID (**C**) mice were analyzed using Bioplanet 2019 with *p*-value ranking in Enrichr, showing the top 10 dominant pathways within each mouse group. Volcano plots showing differentially expressed cytokine (**D**) and chemokine (**E**) genes between the two groups. The transcript levels of each gene were measured by quantitative reverse transcription real-time PCR, and relative gene expression levels were calculated using *Gapdh* as a control gene. (**F**) Summarized results from two groups of mice (n = 7 per group) show statistically significant upregulation of chemokine genes in CB17.SCID lungs compared to those of NOD.SCID mice. (**G**) A summarized graph obtained from the same dataset as (F) shows higher expression of *Cxcl14* chemokine genes in NOD.SCID lungs than in CB17.SCID mice on day 1 post-infection. Statistical significance indicated as follows: *p < 0.05; ***p < 0.001

To identify a molecular signature associated with attenuated lung inflammation in influenza-infected NOD.SCID mice, we extracted mRNAs from the lungs of NOD.SCID and CB17.SCID mice one day after IAV challenge. Bulk RNA sequencing analysis was performed, resulting in 1703 differentially expressed genes (DEGs) out of a total of 20,623 genes. The DEGs were filtered based on a *p*-value < 0.05 and fold change > 2; 827 genes (49%) showed significant downregulation and 876 genes (51%) showed significant upregulation in NOD.SCID mice compared to CB17.SCID mice (Fig. 2A).

To explore the major pathways associated with these DEGs, we conducted pathway analysis using Bioplanet 2019 (*p*-value ranking) in Enrichr (https://maayanlab.cloud/Enrichr/). Through this analysis, we identified the top 10 upregulated (Fig. 2B and Additional file 1: Table S1) and downregulated (Fig. 2C and Additional file 1: Table S2) signaling pathways in NOD.SCID mice. Among them, the cytokine-cytokine receptor interaction was confirmed to be the most prominently downregulated pathway in NOD.SCID mice (Fig. 2D, E). In particular, the early response cytokines type I IFN (*ifna4* and *ifnb1*) and type III IFN (*ifnl2* and *ifnl3*) secreted by pulmonary epithelial cells, alveolar macrophages, and plasmacytoid dendritic cells in response to recognition of IAVs were upregulated in CB17.SCID mice [6]. Notably, the upregulation of IFN-λ (i*fnl2* and *ifnl3*), which is restricted to epithelial cells [6], was significantly reduced in NOD.SCID mice, suggesting that the pulmonary epithelial response to IAV infection was reduced in NOD.SCID mice compared to CB17.SCID mice.

Early inflammatory cytokines such as IL-1 (*Il1a*, *Il1b*), IL-6 (*Il6*) and TNF-α (*Tnf*) [6] were markedly reduced in NOD.SCID mice. Similarly, CCL2 (*Ccl2*), a ligand for CCR2 that recruits CCR2^+^ inflammatory monocytes [13], as well as CXCL1 (*Cxcl1*), CXCL2 (*Cxcl2*), and CXCL5 (*Cxcl5*), which are ligands of CXCR2 that induce neutrophil infiltration in influenza-infected lungs [26–28], showed notable decreases in NOD.SCID mice. Likewise, CXCL11 (*Cxcl11*), a ligand of CXCR3 involved in neutrophil recruitment [29], and CCL20, which promotes the migration of immune cells such as monocytes, eosinophils, and NK cells [30], were also significantly lower in NOD.SCID compared to CB17.SCID mice. The downregulated expression of these chemokine genes in the lungs of NOD.SCID mice were further confirmed by quantitative real-time PCR (Fig. 2F). In contrast to many inflammatory cytokines and chemokines, RNA sequencing analysis revealed an upregulation of some cytokine and chemokine genes, particularly the *Cxcl14* gene, in NOD.SCID mice compared to the CB17.SCID control (Fig. 2E). Quantitative real-time PCR analysis confirmed that *Cxcl14* gene expression was significantly higher in NOD.SCID mice than in CB17.SCID mice (Fig. 2G).

### Protection of mice against lethal influenza infection by inoculation with AAV9-EGFP vector

CXCL14 is a 9.4 kDa chemokine produced by various immune and nonimmune cells, including pulmonary epithelial cells, and involved in diverse functions, such as the modulation of leukocyte migration, differentiation, and antimicrobial activity [31, 32]. Notably, CXCL14 has been reported to inhibit M1 macrophage polarization and proinflammatory cytokine production in a sepsis-associated kidney injury model [33]. This raised the possibility that overexpression of CXCL14 may contribute to the suppression of lung inflammation following influenza infection in NOD.SCID mice.

**Fig. 3:**
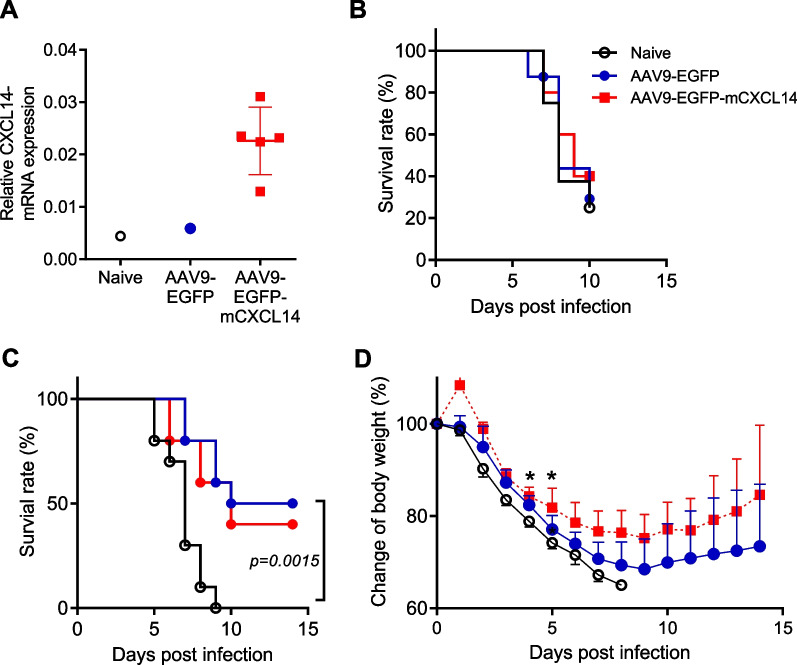
Protective effect of airway inoculation with the AAV9 vector against lethal influenza infection in mice. (**A**) AAV9-EGFP or AAV9-EGFP-mCXCL14 vectors were administered to BALB/c mice, and lung *Cxcl14* gene expression was quantified by qPCR 2 weeks later. Naive mice were used as negative controls. The AAV9-EGFP-mCXCL14 group exhibited upregulated *mCXCL14* gene expression compared to both the naive and AAV9-EGFP groups. (**B**) Two weeks after AAV9 vector inoculation, mice were infected with IAV. Naive mice infected with IAV served as a control. A summarized survival graph of the three groups (n = 5 per group) shows no significant difference between the groups. (**C**-**D**) Mice were infected with IAV 4 weeks after AAV9 vector administration. Cumulative follow-up results from two independent experiments (n = 10 per group) show reduced mortality (**C**) and attenuated weight loss (**D**) in mice receiving AAV9-EGFP or AAV9-EGFP-mCXCL14 vectors compared to infected naive controls. Statistical analysis was performed using the log-rank (Mantel-Cox) test for survival comparison and multiple t-test for weight change comparison. Statistical significance is indicated as follows: *p < 0.05

To investigate this hypothesis, we delivered the *Cxcl14* gene to the lungs of mice using the AAV9-EGFP vector. AAV is known for its nonpathogenicity and low immunogenic nature, enabling long-term expression of therapeutic genes in both animals and humans [15, 16]. Specifically, the AAV9-EGFP vector has recently been utilized to transduce the human ACE2 gene into mouse airway epithelial cells for the development of a SARS-CoV-2 mouse infection model [34]. Based on this, we delivered the AAV9-EGFP control or AAV9-EGFP-mCXCL14 vectors into the lungs of BALB/c mice through oropharyngeal inoculation, followed by intranasal infection with a lethal dose of IAV. As a negative control, mice were also challenged with IAV without receiving the AAV9-EGFP vector. Increased *Cxcl14* gene expression in AAV9-EGFP-mCXC14 inoculated lungs was confirmed by qPCR 2 weeks later (Fig. 3A). After IAV infection, the survival time and rate were compared between mice in these three groups. First, mice were infected with IAV two weeks after AAV9 inoculation according to a previously reported protocol [34]. However, no difference in survival time or rate was observed between these three groups (Fig. 3B).

Next, the time interval between vector inoculation and influenza infection was extended to 4 weeks, considering previous reports that indicated progressively increased expression of AAV-transduced genes for more than 12 weeks after intravenous injection of the vectors [35]. Surprisingly, both mice receiving the AAV9-EGFP control or AAV9-EGFP-mCXCL14 vectors exhibited statistically significant survival rates and prolonged survival times compared to naive control mice infected with IAV. Among the mice receiving AAV9-EGFP and AAV9-EGFP-mCXCL14 vectors, 50% (5/10) and 40% (4/10), respectively, were protected from lethal influenza infection, whereas all naive mice experienced 100% mortality (Fig. 3C). Furthermore, morbidity, as indicated by weight loss, was attenuated in both groups of vector-treated mice compared to the naive control (Fig. 3D).

**Fig. 4:**
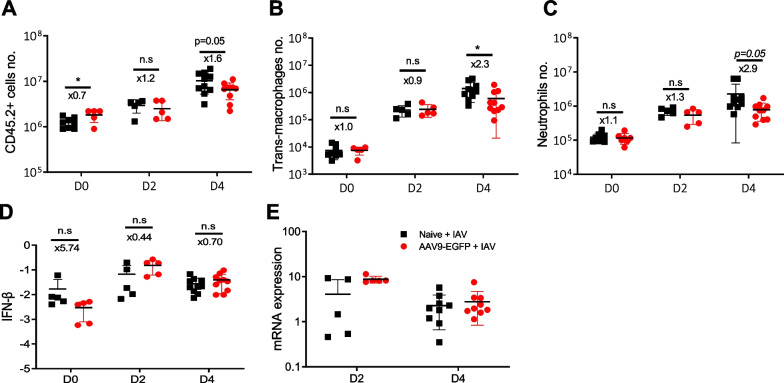
Comparsion of innate cells, cytokines, and virus titers in IAV-infected lungs of naive and AAV9-EGFP vector-treated mice. Cells were isolated from mouse lungs before IAV infection (D0) or on days 2 (D2) and 4 (D4) post-infection, four weeks after AAV9-EGFP vector administration. Absolute numbers of each immune cell were calculated based on flow cytometry analysis: CD45.2^+^ cells (**A**), trans-macrophages (**B**), and neutrophils (**C**). Naive mice infected without pre-administered AAV9 vector were used as controls. Cumulative results from two independent experiments (n = 5–10 per group) show lower numbers of neutrophils and trans-macrophages on day 4 post-infection in the lungs of AAV9 vector-treated mice compared to naive controls. Total mRNA was extracted from mouse lungs before IAV infection (D0) or on days 2 (D2) and 4 (D4) post-infection, four weeks after AAV9-EGFP vector administration. (**D**) IFN- β gene expression levels were quantified by qPCR and normalized to *Gapdh*. Cumulative results from two independent experiments (n = 5–10 per group) showed no significant difference between the two groups. (**E**) Total mRNAs were extracted from the lungs of mice infected with IAV in the presence or absence of pre-administered AAV9 vector. Levels of IAV N gene transcripts were measured by quantitative PCR, with *Gapdh* as a control gene for relative expression. Cumulative results from two independent experiments (n = 5–10 per group) show no difference in IAV gene expression levels in between the two groups. The data are means ± SDs; the x-values are fold changes. Statistical significance is indicated as follows: *p < 0.05

Subsequently, inflammatory cells in the lungs of AAV9-EGFP vector-treated mice and naive control mice were analyzed by flow cytometry prior to IAV infection (day 0), as well as on days 2 and 4 after infection (Fig. 4A). On day 0, the AAV9 vector-treated group showed higher numbers of monocytes (Ly6C^−^ or Ly6C^+^), NK cells, CD8 T cells, and B cells compared to the naive control group (Additional file 1: Fig. S1). However, by day 4 post-infection, a significant difference in the number of trans-macrophages and neutrophils was observed between the two groups (Fig. 4B, C). Notably, infiltration of trans-macrophages and neutrophils into the infected lungs was attenuated in AAV9 vector-treated mice compared to naive mice. Although the number of NK cells remained higher in the AAV9 vector group on day 2 post-infection, no significant differences were observed in other immune cells, including alveolar macrophages, eosinophils and CD4 T cells (Additional file 1: Fig. S1). Furthermore, the gene expression levels of cytokines, including IFN-β, and chemokines were found to be comparable in the lungs of both groups of mice (Fig. 4D and Additional file 1: Fig. S2). Finally, to assess whether AAV9 vector inoculation affected IAV replication or clearance, lung viral titers were measured by qPCR, and no significant difference was found between the two groups (Fig. 4E). Taken together, these results suggest that pulmonary AAV9-EGFP vector inoculation may protect mice against lethal influenza infection by inhibiting the migration of neutrophils and trans-macrophages into the infected lung.

## Discussion

The main goal of this study was to identify strategies to control influenza-associated lung injury and protect the host against lethal influenza infection. Initially, we focused on comparing the innate immune response between CB17.SCID and NOD.SCID mice. Our findings indicated that NOD.SCID mice, which exhibited attenuated infiltration of neutrophils and inflammatory monocytes, experienced reduced morbidity and mortality compared to CB17.SCID mice. Additionally, an increase in *Cxcl14* chemokine gene expression was observed in infected NOD.SCID lungs. Based on these findings, the *Cxcl14* gene was delivered to the lungs of mice using the AAV9-EGFP vector to investigate whether *Cxcl14* overexpression could alleviate inflammatory cell infiltration in IAV-infected lungs. Contrary to expectations, mice receiving the AAV9 vector with the *Cxcl14* gene prior to IAV infection showed similar morbidity in terms of weight loss and mortality compared to mice receiving a control AAV9 vector. However, approximately half of the hosts in both groups survived, whereas all naive mice that did not receive the AAV9 vector succumbed early after IAV challenge. Further analysis revealed that AAV9 vector inoculation into the respiratory tract resulted in reduced infiltration of neutrophils and trans-macrophages in the infected lungs.

When BALB/c, CB17.SCID, and NOD.SCID mice were infected with IAV, and only BALB/c mice were able to recover after initial weight loss, while both CB17.SCID and NOD.SCID, lacking adaptive immune cells, failed to survive due to inadequate virus control, as expected. Furthermore, there was no significant difference in weight loss between BALB/c and CB17.SCID mice during the first 5 days, when adaptive immunity might not be fully activated to participate in virus control [6]. However, NOD.SCID mice exhibited a slower decline in body weight and longer survival compared to CB17.SCID mice, despite similar virus titers in all three groups. Flow cytometric analysis revealed that exaggerated infiltration of neutrophils and inflammatory monocytes was associated with severe morbidity and shortened survival in CB17.SCID mice. These findings support previous reports suggesting that attenuation of neutrophil and monocyte infiltration in influenza-infected lungs has beneficial effects on host morbidity and mortality [11, 13, 14].

One of the notable findings from the initial experiments in this study was that an inverse relationship was identified between the expression level of the *Cxcl14* gene and the degree of inflammatory cell influx in infected lungs. CXCL14 has been previously suggested to have an anti-inflammatory effect [33, 36]. However, TNF-α can downregulate *Cxcl14* expression [37], and the absence of CXCL14 does not affect the severity of IAV infection [38]. In our current study, we observed much higher levels of TNF-α in severely inflamed CB17.SCID lungs in contrast to NOD.SCID lungs. We also found that the delivery of the *Cxcl14* gene using AAV9 vectors into the mouse airway failed to demonstrate beneficial effects of CXCL14 chemokine on the survival of IAV-infected hosts. These results suggest that the elevated expression of *Cxcl14* in the infected lungs of NOD.SCID mice might be a consequence of reduced TNF-α levels, rather than indicative of the anti-inflammatory effect of this chemokine.

In this study, the administration of AAV9 vectors containing only EGFP, which are commonly used as control vectors in gene transfer experiments with AAV9 vectors, showed a prophylactic effect against lethal influenza infection. Interestingly, this effect was observed only when the AAV9 vector was inoculated 4 weeks prior to IAV infection, not 2 weeks. A previous report demonstrated that intranasal instillation of replication-deficient adenovirus (E1 & E3 gene-deleted Ad5 empty, AdE) particles without a transgene also protected BALB/c mice against lethal IAV infection [39]. However, AdE particles were administered 2 days prior to IAV challenge, and their effect was attributed to the rapid induction of inflammatory cytokines/chemokines and the antiviral response, although the specific mechanisms were not well understood. In contrast to adenovirus particles, AAV vectors are known not to induce acute inflammation [16]. Moreover, the main feature observed in AAV9-treated mice in the current study was the attenuated infiltration of neutrophils and trans-macrophages in IAV-infected lungs, suggesting that the prophylactic mechanisms of AAV9 and AdE may be different.

Although AAV vectors are generally considered to have low immunogenicity, their immunogenicity can be dose dependent, and adaptive immune responses against the transgene product and viral cap protein can limit their long-term efficacy [16, 40]. Moreover, the double-stranded DNA (dsDNA) vector genome can trigger innate immunity [40]. Notably, a previous study demonstrated that dsRNA transcribed from viral dsDNA can promote IFN-β production in host cells [20]. This IFN-β can suppress AAV transgene expression in vivo and in vitro, and the production of IFN-β in host cells is dependent on the MDA5 dsRNA sensor, suggesting that dsRNA can trigger IFN-β production. This possibility was further confirmed by the detection of transgene-derived minus strand transcripts, which are essential for dsRNA formation. Interestingly, the level of IFN-β induction in humanized mice was higher at 8 weeks compared to 4 weeks after injection of the AAV vector carrying the human Factor IX gene [20]. On the other hand, other research has shown that transgene expression levels progressively increased after gene delivery using AAV vectors, especially for more than 12 weeks in mice [35], suggesting that the levels of IFN-β induction correlate with transgene expression levels. These previous findings can explain why the prophylactic effect of the AAV9 vector was observed at 4 weeks rather than 2 weeks after vector administration, as observed in the current study.

This study has certain limitations in terms of fully understanding the prophylactic mechanism of the AAV9 vector. The specific mechanisms involved were not thoroughly documented in the current study. While the increased infiltration of monocytes, NK cells and CD8 T cells in the lungs of AAV9-inoculated mice may contribute to early viral control, no significant difference in viral gene expression levels was observed between the AAV9 and control groups. Alternative explanations could include IFN-β production triggered by dsRNA generated from the transgene, which may induce antiviral genes in the alveolar epithelium, the primary host cell of the AAV9 vector. Another possibility is that long-term exposure to low levels of IFN-β may lead to desensitization of innate immune cells. However, no significant increase in IFN-β gene expression was observed in AAV9 vector-treated mice compared to naive control mice. To re-evaluate this finding, it is crucial to assess the expression of the IFN-β gene specifically in isolated lung epithelial cells and investigate the potential elimination of the protective effect of the AAV9 vector by blocking IFN-β activity. Additionally, conducting studies that deplete neutrophils or monocytes could clarify whether the presence of these cells is necessary for the protective effect of the AAV vector.

## Conclusions

This study demonstrates a potential strategy for controlling lung immunopathology following severe influenza infection. To control lung inflammation, BALB/c mice were infected with IAV following AAV9 inoculation, which resulted in attenuated infiltration of trans-macrophages, neutrophils and monocytes in the lungs. These findings imply that pre-inoculation with AAV9 prior to IAV infection protected mouse lungs from immunopathology by reducing the recruitment of inflammatory cells. Although there was no difference in lung viral titers between the naive group and the AAV9 pre-inoculated group, this study represents the first report demonstrating the protective effect of the AAV vector in a severe influenza infection model.

## Methods

### Animals

BALB/c, NOD.SCID and CB17.SCID mice were obtained from Central Lab. Animal Inc. (Seoul, South Korea). These mice were housed in sterilized cages with access to sterilized food and water at the laboratory animal facility of the Biomedical Center for Animal Resource Development at Seoul National University, as well as the Biomedical Research Institute of Seoul National University Hospital (Seoul, South Korea). The mice used in this study were 6 weeks old with an average body weight of 18 g. Ethical approval for this study was obtained from the Institutional Animal Care and Use Committee (IACUC) of the Seoul National University Institute of Laboratory Animal Resource (IACUC Number: 220221-3-2) and the Biomedical Research Institute of Seoul National University Hospital (IACUC number: 22-0235-S1A1). All experimental procedures were conducted in compliance with international and institutional guidelines.

### Experimental group and monitoring

To assess the severity of IAV infection, BALB/c, CB17.SCID, and NOD.SCID mice were intranasally infected with IAV. CB17.SCID mice and NOD.SCID mice were infected with IAV and sacrificed on days 1, 3, and 5 for flow cytometry analysis. CB17.SCID and NOD.SCID mice were used for bulk RNA sequencing analysis 1 day after IAV infection. Daily weight measurements were recorded for all mice after IAV infection. For the AAV9 vector experiment, BALB/c mice were divided into three groups as follows: the naive group received no treatment, group 2 was inoculated with AAV9-EGFP only, and group 3 was inoculated with AAV9-EGFP-mCXCL14 containing the murine *Cxcl14* gene. Subsequently, these mice were infected with IAV 2 weeks later. Second, BALB/c mice were divided into two groups as follows: the naive group received no treatment, and group 2 was inoculated with AAV9-EGFP only. Subsequently, these mice were infected with IAV 4 weeks later. Daily weight measurements were recorded for all mice after IAV infection. All mice were sacrificed 0, 2, and 4 days after IAV infection to analyze inflammatory cell infiltration and gene expression in the infected lungs.

### Virus preparation, AAV production, infection, and tissue harvest

The influenza virus strain A/PR/8/34 (PR8, H1N1) was used in this study. Viral stocks were obtained by transfecting MDCK cells, as provided by Pr. YK Choi’s laboratory (Chungbuk National University, Chungbuk, South Korea). The virus was harvested 24 h after transfection and stored at − 80 °C until use. For infection, mice were anesthetized with isoflurane and oxygen. Intranasal infection was performed by administering 50 μl of phosphate buffered saline (PBS) containing IAV at a dose of 3.5 × 10^5^ PFU/ml, ensuring deposition of the virus in the lower respiratory tract. AAV9-EGFP and AAV9-EGFP-mCXCL14 vectors were purchased from Vector Builder (Chicago, Illinois, USA) as 2 × 10^13^ stocks and administered to mice via oropharyngeal delivery under anesthesia. A bolus inoculation of 50 µl of AAV9-EGFP at a dose of 10^11^ GC (genome copies) per mouse was given using a pipette. Infection procedures were performed in a biosafety level 2 laboratory in accordance with the animal facility guidelines of the Biomedical Research Institute of Seoul National University Hospital. Lung tissues were harvested with PBS and cut using a razor. Dissociation medium was prepared by adding 50 μl of collagenase type IV (0.1 g/ml, Worthington Biochemical Corporation, NJ, USA) and 50 μl of DNase I solution (1 mg/ml, Sigma, St. Louis, Missouri, USA) to 50 ml of RPMI 1640 medium and incubated at 37 °C for 2 h on a shaking platform.

### Preprocessing and analysis of bulk RNA-Seq data and gene enrichment

The infected lungs from euthanized mice were homogenized with DMEM, and lung tissue was used for RNA isolation using a BIO-52072 RNA isolation kit from Meridian Bioscience (Memphis, TN, USA). Next-generation sequencing (NGS) was performed using the Illumina NovaSeq platform at Macrogen (Seoul, South Korea). The clean reads obtained were mapped to the genome, and transcripts were assembled and merged using StringTie v2.1.3b with corresponding genome annotation. Genes with a *p* value less than 0.05 and a fold change greater than 2 were selected for gene set enrichment analysis in Enrichr (https://maayanlab.cloud/Enrichr/).

### Flow cytometry analysis

Mononuclear cells were isolated from the lungs of infected or uninfected mice. The samples were stained with specific monoclonal antibodies including Ly6G (1A8), CD11b (M1/70), CD11c (N418), CD49b (DX5), F4/80 (BM8), CD45.2 (104), CD64 (X54-5/7.1), Siglec F (E50-2440), and Ly6C (HK1.4). These antibodies were obtained from BD Bioscience (San Jose, CA, USA), eBioscience (San Diego, CA, USA), and BioLegend (San Diego, CA, USA). The prepared cells were resuspended in staining buffer (PBS, 0.5% bovine serum albumin, and 0.5 mM EDTA), and single-cell suspensions were labeled with antibodies for 30 min at 4 °C. Flow cytometry analysis was performed using an LSRFortessa X-20 instrument from BD Bioscience, and the acquired data were analyzed using FlowJo software v10 from TreeStar (San Carlos, USA).

### Quantitative real-time PCR

Lung tissue was processed to extract total RNA using the Total RNA Isolation Kit (BIO-52072, Meridian Bioscience) following the manufacturer’s instructions. cDNA synthesis was performed using AccuPower Cycle Script RT PreMix (Bioneer) with the extracted RNA. The expression levels of the IAV gene (NS), CCL2, CCL20, CXCL1, CXCL2, CXCL5, CXCL11, CXCL14, and glyceraldehyde-3-phosphate dehydrogenase (GAPDH) were assessed by qPCR analysis. The sequences of primers used for qPCR can be found in Additional file 1: Table S3. The qPCR included 1 µl of cDNA (0.5 μg/μl), 1 µl of each primer, and 5 µl of PowerUp SYBR Green Master Mix (A25742, Applied Biosystems, Framingham, MA, USA). qPCR was performed using a CFX Connect real-time PCR instrument (Applied Biosystems). The PCR program consisted of an initial incubation at 95 °C for 10 min, followed by 40 cycles of 15 s at 95 °C and 60 s at 60 °C, and a final step of 95 °C for 15 s, 60 °C for 60 s, and 95 °C for 15 s. The gene expression levels were quantified using SYBR-Green, and the relative expression of RNA was normalized to the internal control GAPDH.

### Statistical analysis

Statistical analysis was conducted using GraphPad Prism version 8.0 (GraphPad Software Inc., San Diego, CA, USA). The data are presented as the mean of experimental measurements with standard deviation (SD). Statistical significance was determined using the t-test, and *p* values less than 0.05 were considered statistically significant.

### Supplementary Information

**Additional file 1: Table S1**. Top 10 upregulated signaling pathways in NOD.SCID mice vs. CD17.SCID mice lungs.** Table S2**. Top 10 downregulated signaling pathways in NOD.SCID mice vs. CD17.SCID mice lungs.** Table S3**. Sequences of primers (mouse) used for qPCR. **Fig. S1**. Comparison of immune cells in IAV-infected lungs of AAV9-EGFP vector-treated and naive control mice.** Fig. S2**. Comparison of cytokine and chemokine gene expression in IAV-infected lungs of AAV9-EGFP vector-treated and naive control mice.

## Data Availability

The datasets used and/or analyzed during the current study are available from the corresponding author on reasonable request.

## Abbreviations

AAV: Adeno- associated virus
Cap: Capsid
cDNA: Complementary DNA
DEG: Differentially expressed genes
dsRNA: Double stranded RNA
EGFP: Enhanced green fluorescent protein
GC: Genomic copies
HA: Hemagglutinin
IAV: Influenza A virus
IFN: Interferon
ITR: Inverted terminal repeats
mCXCL14: Murine-CXCL14
NA: Neuraminidase
PBS: Phosphate buffered saline
qPCR: Reverse transcription quantitative real-time PCR
Rep: Replication
TCR: T-cell receptor
