# Supplementary material for: Prevention of severe lung immunopathology associated with influenza infection through adeno-associated virus vector administration
Source: Lab Anim Res. 2023 Oct 30;39:26. doi: 10.1186/s42826-023-00177-0 (PMC10614381; doi:10.1186/s42826-023-00177-0)
Supplement: Supplementary file 1 — Additional file 1: Table S1. Top 10 upregulated signaling pathways in NOD.SCID mice vs. CD17.SCID mice lungs. Table S2. Top 10 downregulated signaling pathways in NOD.SCID mice vs. CD17.SCID mice lungs. Table S3. Sequences of primers (mouse) used for qPCR. Fig. S1. Comparison of immune cells in IAV-infected lungs of AAV9-EGFP vector-treated and naive control mice. Fig. S2. Comparison of cytokine and chemokine gene expression in IAV-infected lungs of AAV9-EGFP vector-treated and naive control mice. [file 42826_2023_177_MOESM1_ESM.pdf]

Prevention of severe lung immunopathology associated with influenza infection through adeno-associated virus vector administration

Eun Ah Choi[1], Hi Jung Park[1], Sung Min Choi[1], Jae Il Lee[2,5,*], and Kyeong Cheon Jung[2,3,4,*]

Supplementary Table

Table S1. Top 10 upregulated signaling pathways in NOD.SCID mice vs. CD17.SCID mice lungs.

Index	Name	P-value	Adjusted p-value	Odds Ratio	Combined score	Gene list
1	Cytokine-cytokine receptor interaction	4.346E-10	1.217E-07	3.11	67.09	CXCL1,2,3,5,9,10,14, CCL2,3,11,20, IL-1α,1β,6
2	Viral protein interaction with cytokine and cytokine receptor	1.314E-08	1.840E-06	4.85	87.97	CCR1,4, CXCL1,2,3,5,9,10,14, CCL2,3,4,7,11,20,22,24, IL-6, CXCR1,2
3	IL-17 signaling pathway	4.456E-07	4.159E-05	4.38	63.99	CXCL1,2,3,5,10, CCL2,7,11,20, MMP3, FOSL1, MAPK10, IL-6,1β
4	Rheumatoid arthritis	6.890E-06	4.381E-04	3.88	46.1	MMP3, CXCL1,2,3,5, CCL2,3,20, IL-1α,1β,6, IFNG,CTSK
5	TNF signaling pathway	7.824E-06	4.381E-04	3.52	41.34	CXCL1,2,3,5,10, CCL2,20, MAPK1, SOCS3, IL-6,1β, IFNβ1, MMP3
6	NOD-like receptor signaling pathway	6.245E-05	2.914E-03	2.59	25.12	CXCL1,2,3, CCL2, GBP2,3,5,7, IFNβ1, STAT2, IL-6, NLRP12 ,IL-1β, MYD88
7	Hepatitis C	3.655E-04	1.409E-02	2.49	19.74	IFNA4,CDKN1A,RSAD2,IFNβ1,STAT2,IFIT1,SOCS3,CLDN4,6,OAS2,3
8	Chemokine signaling pathway	4.034E-04	1.409E-02	2.31	18.05	CXCL10,IFNA4,IFNβ1,STAT2,IFIT1,SOCS3,CLDN4,6,IAS2,3
9	Dilated cardiomyopathy	4.746E-04	1.409E-02	2.98	22.84	MYBPC3,RYR2,LAMA1,TNNC1,ADCY4,PLN,ACTC1,SGCA,TNNT2
10	Influenza A virus	5.032E-04	1.409E-02	2.37	17.99	MYBPC3,RYR2,LAMA1,TNNC1,TNF,IL-6,ACTC1,SGCA,TNNT2,MYL3

Table S2. Top 10 downregulated signaling pathways in NOD.SCID mice vs. CD17.SCID mice lungs.

Index	Name	P-value	Adjusted p-value	Odds Ratio	Combined score	Gene list
1	Dilated cardiomyopathy	0.0002788	0.04352	3.46	28.28	MYBPC3,RYR2,LAMA1,TNNC1,PLN,ACTC1,SGCA,TNNT2,MYL3,TNNI3,SGCG,MYH6,CACNG4
2	Cardiac muscle contraction	0.0003987	0.04352	3.53	27.61	MYL4,RYR2,ACTC1,TNNC1,TNNT2,MYL3,CASQ2,HRC,TNNI3,MYH6,CACNG4,TRDN
3	Hypertrophic cardiomyopathy	0.000563	0.04352	3.39	25.48	MYBPC3,RYR2,ACTC1,SGCA,LAMA1,TNNC1,TNNT2,MYL3,TNNI3,SGCG,MYH6,CACNG4
4	ECM-receptor interaction	0.005078	0.3034	2.82	14.9	GP9,GP1BB,LAMA1,CHAD,COL4AA6,TNR,GP1BA,HMMR,GP6,FREM2
5	Adrenergic signaling in cardiomyocytes	0.01474	0.7045	2.09	8.8	RYR2,TNNC1,CALML4,MYL4,PLN,ACTC1,PPP2R2C,TNNT2,MYL3,TNNI3,SCN5A,MYH6,CACNG4
6	Mucin type O-glycan biosynthesis	0.07113	1	2.74	7.23	GALNT6,GALNT13,ST6GALNAC3,B3GNT3
7	Arrhythmogenic right ventricular cardiomyopathy	0.121	1	1.85	3.91	RYR2,SGCA,ACTN2,LAMA1,SGCG,CACNG4
8	Dopaminergic synapse	0.1256	1	1.6	3.33	MAPK10,KCNJ5,PPP2R2C,KIF5C,GNG8,CALML4,GRIN2B,SLC18A2,KCNJ3
9	Circadain entrainment	0.133	1	1.7	3.44	RYR2,KCNJ5,GNG8,CALML4,GRIN2B,RYR3,KCNJ3
10	Protein digestion and absorption	0.1656	1	1.6	2.87	COL17A1,COL26A1,CPB1,KCNE3,ELN,COL4A6,COL10A1

Table S3. Sequences of primers (mouse) used for qPCR

Primer	Forward (5'-3')	Reverse (5'-3')
NS	CAGGACATACTGATGAGGATG	GTTTCAGAGACTCGAACTGTG
CCL2	CCGGCTGGAGCATCCACGTGT	TGGGGTCAGCACAGACCTCTCTCT
CCL20	CGACTGTTGCCTCTCGTACA	GAGGAGGTTCACAGCCCTTT
CXCL1	CACAGGGGCGC CTATCGCCAA	CAAGGCAAGCCTCGCGACCAT
CXCL2	ACCCCACTGCGCCCAGACAGAA	AGCAGCCCAGGC TCCTCCTTTCC
CXCL5	GCATTTCTGTTGCTGTTCACGCTG	CCTCCTTCTGGTTTTTCAGTTTAGC
CXCL11	GGCTTCCT TATGTTCAAACAGGG	GCCGTTACTCGGGTAAATTACA
CXCL14	CCAAGATTCGCTATAGCGAC	CCTGCGCTTCTCGTTCCAGG
GAPDH	TCACCACCATGGAGAAGGC	GCTAAGCAGTTGGTGGTGCA

Supplementary Figure

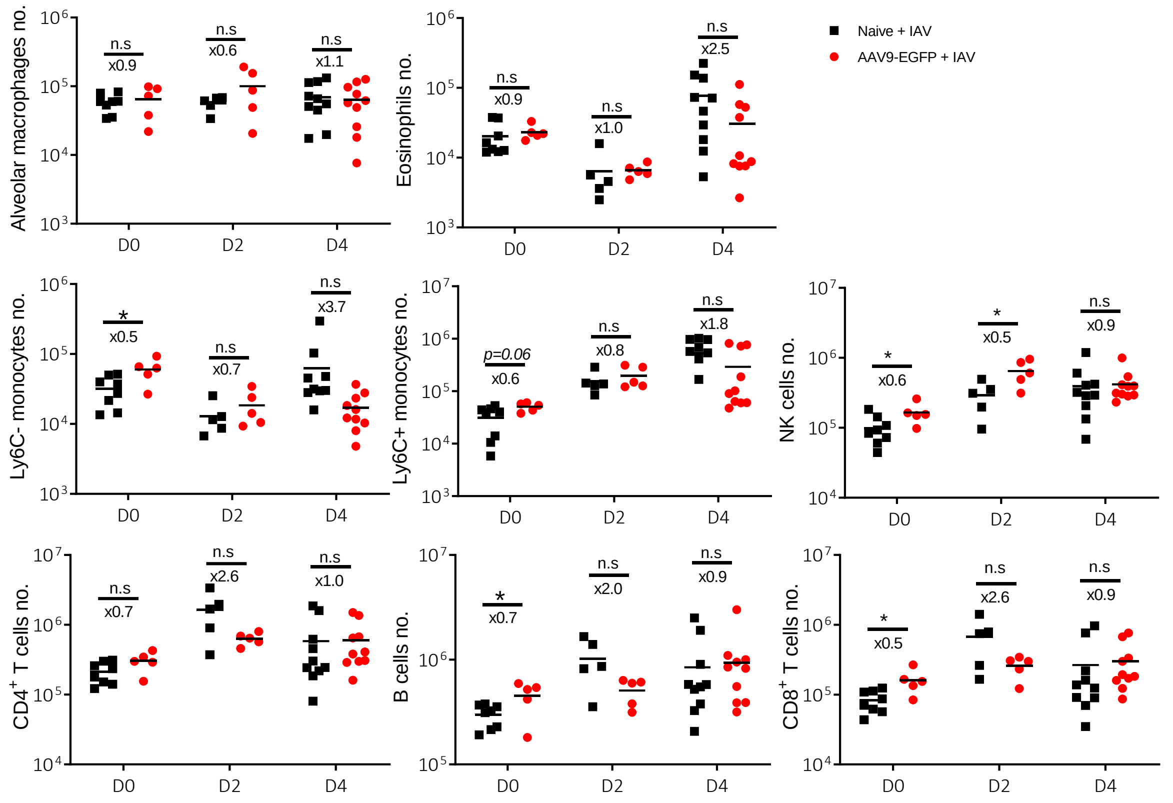

Supplementary Figure S1. Comparison of immune cells in IAV-infected lungs of AAV9-EGFP vector-treated and naive control mice. Cells were isolated from mouse lungs before (D0), on days 2 (D2), and 4 (D4) post-infection with IAV, following four weeks of AAV9-EGFP vector administration. The absolute numbers of each immune cell type were determined through flow cytometry analysis. Naive mice infected without pre-administered AAV9 vector served as controls. The data represent the mean ± SDs from two independent experiments (n= 5 ~10 per group); the x-values are fold changes. Statistical significance is denoted as follows: *, $p < 0.05$; n.s, not significant.

Supplementary Figure

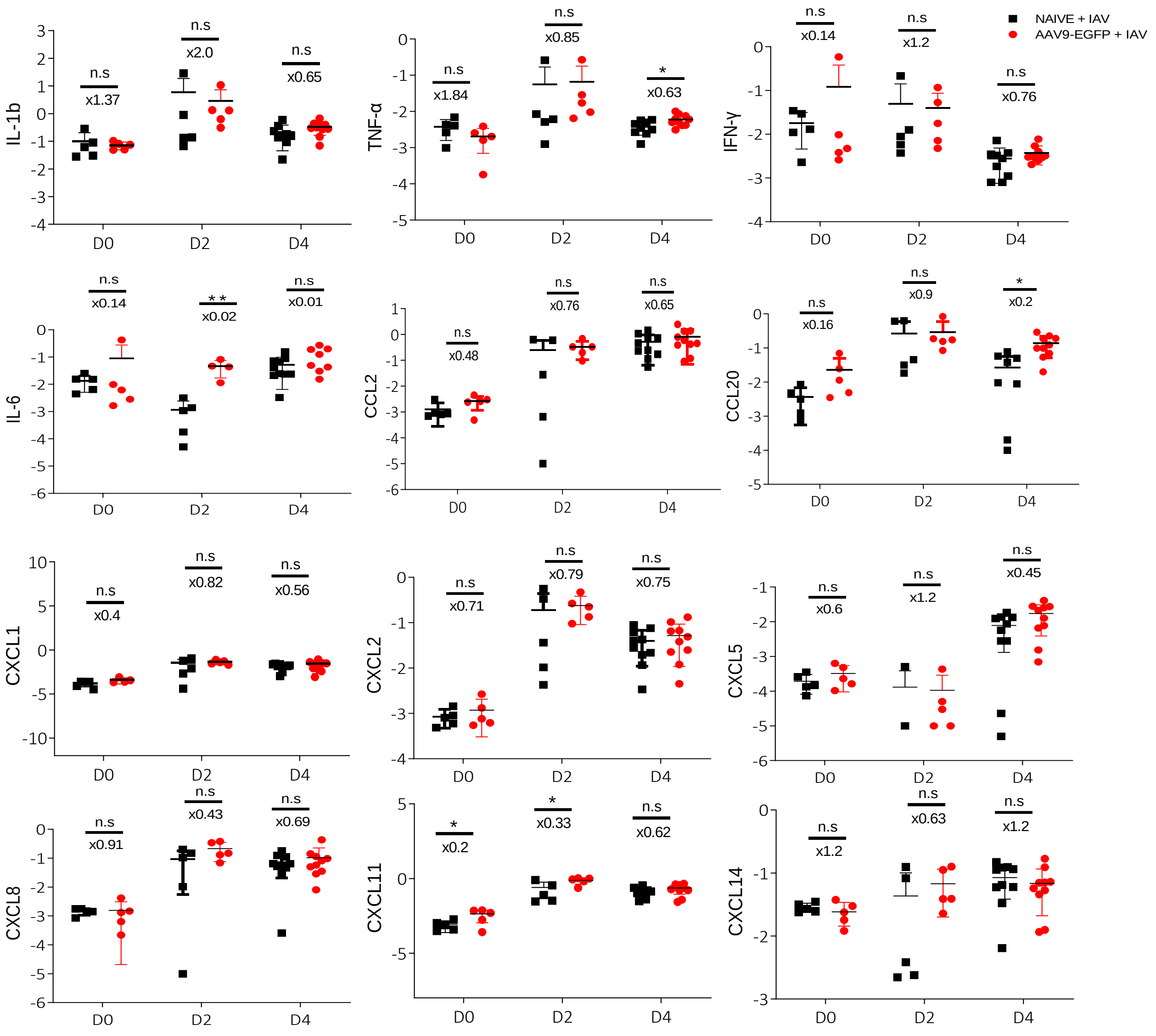

Supplementary Figure S2. **Comparison of cytokine and chemokine gene expression in IAV-infected lungs of AAV9-EGFP vector-treated and naive control mice.** Total mRNA was extracted from mouse lungs before (D0), on days 2 (D2), and 4 (D4) post-infection with IAV, following four weeks of AAV9-EGFP vector administration. Relative levels of cytokine and chemokine gene expression were quantified using qPCR, with normalization to the Gapdh gene. Naive mice infected without pre-administered AAV9 vector were used as controls. The data represent the mean ± SDs from two independent experiments (n= 5 ~10 per group); the x-values are fold changes. Statistical significance is denoted as follows: *, $p < 0.05$; **, $p < 0.01$; n.s, not significant.
